# The Significance of Serum S100A9 and TNC Levels as Biomarkers in Colorectal Cancer

**DOI:** 10.7150/jca.31267

**Published:** 2019-08-29

**Authors:** Minze Zhou, Maoyu Li, Xujun Liang, Ye Zhang, Huichao Huang, Yilu Feng, Guoqiang Wang, Ting Liu, Zhuchu Chen, Haiping Pei, Yongheng Chen

**Affiliations:** 1 Key Laboratory of Cancer Proteomics of Chinese Ministry of Health, Xiangya Hospital, Central South University, Changsha 410008, China; 2 Department of Gastrointestinal Surgery, Xiangya Hospital, Central South University, Changsha, China.; 3 Department of Gastroenterology, Xiangya Hospital, Central South University, Changsha 410008, China.

**Keywords:** S100A9, Tenascin-c, colorectal cancer, serum biomarker

## Abstract

**Purpose**: The aim of this study was to evaluate the diagnostic value of S100A9 and tenascin-c (TNC) levels as colorectal cancer (CRC) biomarkers in several ways, including through screening tests, differentiation tests, combination with existing biomarkers (CEA and CA19-9), and serum level measurements before and after surgery.

**Materials and Methods**: In this case-control study, S100A9 and TNC serum levels were measured in 460 participants: 258 CRC patients, 99 patients with benign colonic disease (BCD) and 103 healthy donors (HD).

**Results**: The serum levels of S100A9 were 22.32 (14.88-29.55) ng/ml, 10.02 (5.83-14.15) ng/ml and 10.05 (7.68-15.34) ng/ml in the CRC, BCD and HD groups, respectively. The serum levels of TNC were 4.30 (2.12-6.04) ng/ml, 1.60 (1.06-2.30) ng/ml and 2.00 (1.37-3.00) ng/ml in the CRC, BCD and HD groups, respectively. Significantly higher levels of both biomarkers (S100A9 and TNC) were found in CRC patients (both p<0.001).

Both S100A9 and TNC levels were superior to CEA and CA19-9 levels as CRC diagnostic biomarkers; the combination of S100A9, TNC and CEA levels was an excellent biomarker with 79.8% sensitivity and 89.6% specificity. The serum levels of S100A9 and TNC in CRC patients were significantly lower after surgery than before surgery (p<0.01).

**Conclusion**: S100A9 and TNC levels could serve as diagnostic biomarkers of colorectal cancer.

## Introduction

Colorectal cancer (CRC) ranks as the third most common cancer and one of the leading causes of cancer-related death globally 1. The incidence and mortality rates of CRC have continued to rise in China over the past few decades 2, 3, and the rate of CRC in China is much higher than that in the West 4. The overall 5-year survival rate of CRC patients is 66%, but the survival rate of CRC patients who are diagnosed at advanced stages is only 10%-30%. Unfortunately, more than 63% of patients with CRC are diagnosed at the late stage; the early stage diagnosis rate is less than 37% 5. In the clinic, many CRC patients remain asymptomatic during the cancer's silent progression; in contrast, benign polyps and colon inflammation can also cause changes in bowel habits, chronic pain, and hematochezia 6, increasing the difficulty of diagnosing CRC. Colonoscopy is considered the gold standard test for CRC, but this test process is highly invasive by nature; thus, compliance is not high in the general population 7. By far, the most commonly used serum biomarkers for the diagnosis of CRC are CEA and CA19-9, but a study showed that CEA had 33% sensitivity and CA19-9 had only 11% sensitivity in a phase II trial 8. It is necessary to look for ideal serum markers to diagnose or screen CRC early.

In our previous report, stromal proteomes from different stages of CRC were compared using a combination of laser capture microdissection (LCM), iTRAQ labeling and two-dimensional liquid chromatography-tandem mass spectrometry (2D LC-MS/MS) 9. Finally, from the identified 1966 proteins, 222 proteins presenting a significant fold change were quantified in different stages. Through the differentially expressed proteins (DEPs) cluster and path analysis, we found that S100A9 and tenascin-C have the potential to become new serum tumor biomarkers 9. S100A9 is a member of the s100 protein family, which includes at least 21 members 10. As calcium binding proteins, members of the s100 protein family have been reported to be associated with the occurrence and development of many cancers, including pancreatic, ovarian and lung cancers 11. However, S100A9 expression levels in the serum of CRC patients and the associations between S100A9 expression and clinical features have seldom been reported. Tenascin-C (TNC) is the founding member of the tenascin gene family 12, and it was first reported in glioblastomas and has since been widely reported in head and neck neoplasms 13. In vitro studies have shown that tenascin-C expression can affect cell behavior in many ways 14, and our unpublished studies in quantitative proteomics and cytology have been used to validate TNC expression as a novel biomarker in CRC. However, the expression and application of TNC lack the further clinical testing needed to verify its role in CRC patients.

In this study, we collected a large number of clinical serum samples to explore the potential of S100A9 and TNC levels as early diagnostic biomarkers in CRC by using quantitative ELISA. The study also assessed whether S100A9 and TNC levels have the potential to improve the efficiency of diagnosis when combined with other tumor biomarkers in CRC patients.

## Methods and Materials

### Clinical samples

A total of 460 samples were obtained between July 2017 and March 2018 from the Department of Digestive Surgery and the Department of Gastroenterology of Xiangya Hospital, Center South University, China. All samples were classified into the following groups: patients with CRC (n=258), patients with benign colonic disease (including colon polyps and inflammatory bowel disease) (n=99), and healthy donors (n=103). In addition to the above 460 samples, 24 serum samples taken from 21 post-surgery or relapsed patients were used to determine how the serum concentrations of S100A9 and TNC change during disease progression.

CRC patients in the study never received preoperative radiotherapy, chemotherapy, or chemoradiotherapy. Those who had benign colonic disease including infections, collagen diseases, bowel perforation or other debilitating diseases were also excluded. Recruited patients with benign colonic disease (BCD) could have colon polyps and inflammatory bowel disease. Normal serum samples were collected during health examinations from patients with no clinical evidence of CRC, BCD or other cancer. This study was approved and monitored by the ethics committee of Xiangya Hospital, Center South University. Information about the age, sex, and other clinicopathologic features of the participants is shown in Table 3.

### Assay for the determination of S100A9 and TNC levels

A total of 5 ml of venous blood were collected by venipuncture, and the blood was allowed to clot for 2 h at room temperature before being centrifuged for 15 min at 1000 g. The serum was collected after centrifugation, and the assay was run immediately. The concentrations of S100A9 and TNC were measured in duplicate with ELISAs performed according to the procedure from CUSABIO (Catalog Number: S100A9 CSB-E11834h and TNC CSB-E13125h). The plates were read by a multi-detection microplate reader at 450 nm. To correct for any measurement errors within a batch, we revised the data with a standard curve run on each plate and tried to keep the same conditions and external environment for each assay.

### Assay for the determination of CEA and CA19-9 levels

CA19-9 and CEA serum levels were measured by microarray chemiluminescent immunoassay (SUNLANT). Data were collected by the supporting instruments. Using different concentration calibration products as samples, the gray-concentration quantitative standard curve was drawn. The corresponding concentrations of CA19-9 and CEA in serum samples were calculated from the standard curve. The working range of the CA19-9 immune assay is 2-1200 U/ml, and that of the CEA immune assay is 0.1-1000 ng/mL.

### Statistical analysis

Statistical analysis was carried out using SPSS version 24.0 for Windows (Chicago, IL, USA), MedCalc version 18.2 (Ostend, Belgium) and GraphPad Prism version 5.01 (San Diego, CA). Continuous variables with normal distributions were presented as the mean ± standard deviation (SD); nonnormal variables were reported as the median (interquartile range). The receiver operating characteristic (ROC) curve was drawn to assess diagnostic performance. We assessed the discriminatory power by the area under the curve (AUC) and 95% confidence interval. Multivariate analysis used logistic regression models to identify new parameters and obtain new ROC combination biomarkers and AUC values. Univariate comparisons between groups (cases and controls) were performed using chi-square tests or Fisher's exact tests for categorical data and using the Mann-Whitney U test or Kruskal-Willis H test for continuous variables. A two-tailed p value < 0.05 was considered statistically significant.

## Results

### Performance of S100A9 and TNC levels as screening biomarkers in CRC

**Fig 1:**
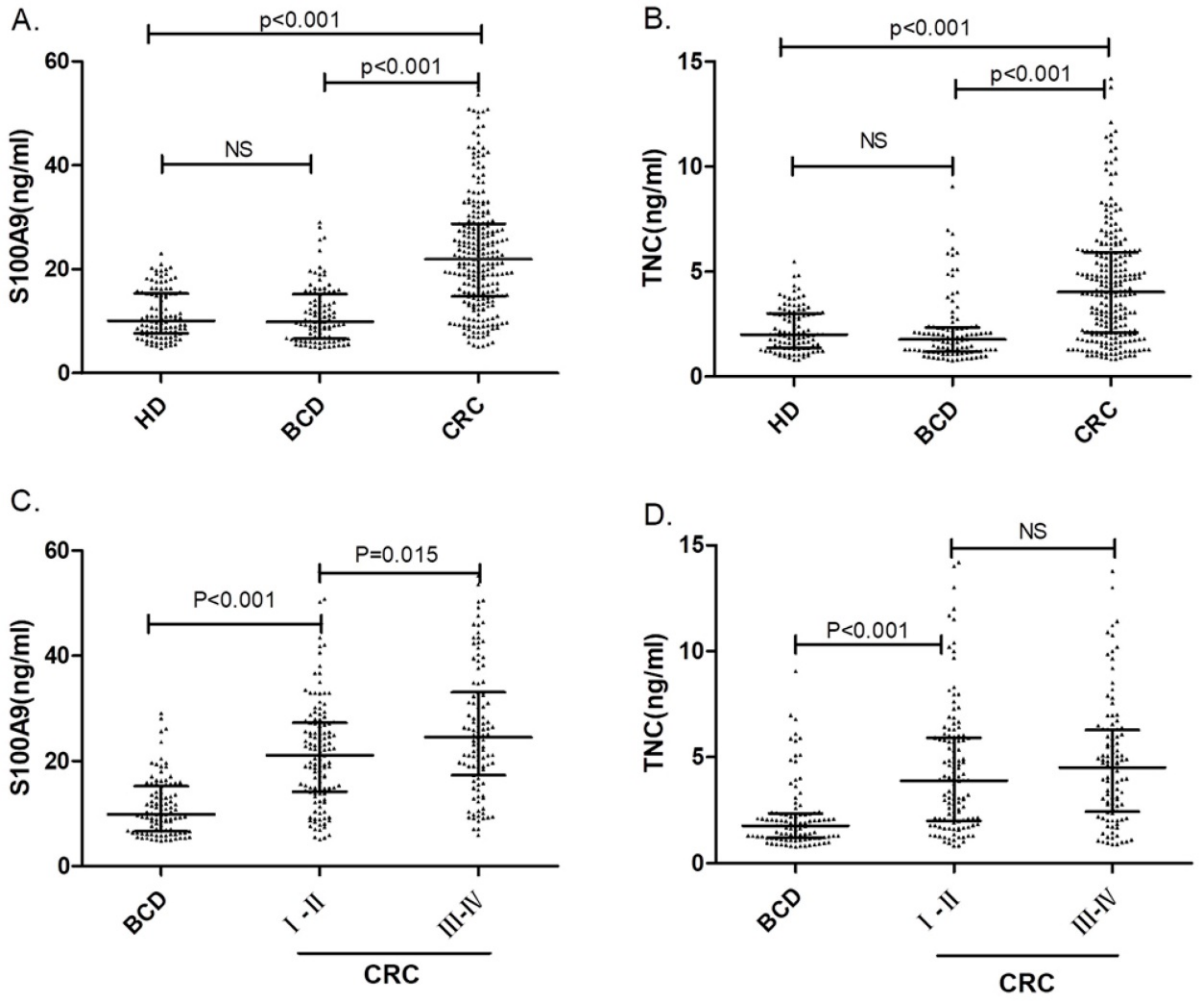
Serum S100A9 and TNC concentrations in colorectal cancer patients, benign colonic disease patients and healthy donors. The comparison of serum S100A9 (A) or TNC (B) levels in CRC patients, benign colonic disease (BCD) patients and healthy donors are shown as dot plots, and the middle line represents the median (p<0.001). Panels (C) and (D) show S100A9 and TNC levels, respectively, in BCD patients and patients with different CRC stages. The three lines in each scatter plot indicate the median and quartiles of this set of data. Abbreviations: CRC: colorectal cancer patients BCD: benign colonic disease patients HD: healthy donors NS: not statistically significant

A total of 103 serum samples from the HD group and 258 serum samples from the CRC patient group were tested by S100A9 and TNC ELISAs. The S100A9 serum concentrations were 10.05 (7.68-15.34) ng/mL and 22.32 (14.88-29.55) ng/mL, respectively. We used Mann-Whitney U test for the unpaired samples, and Median expression levels have be indicated in Fig. 1. As shown in Fig. 1a, the S100A9 serum level in CRC patients was significantly higher than that in healthy donors (p<0.001). Similar results can be found for the TNC serum levels. The concentrations of TNC in the HD and CRC groups were 2.00 (1.37-3.00) ng/mL and 4.30 (2.12-6.04) ng/mL, respectively. The serum level of TNC was remarkably higher in the CRC patient group than in the HD group (p<0.001). Both S100A9 and TNC levels have the potential identify colorectal cancer.

**Fig 2:**
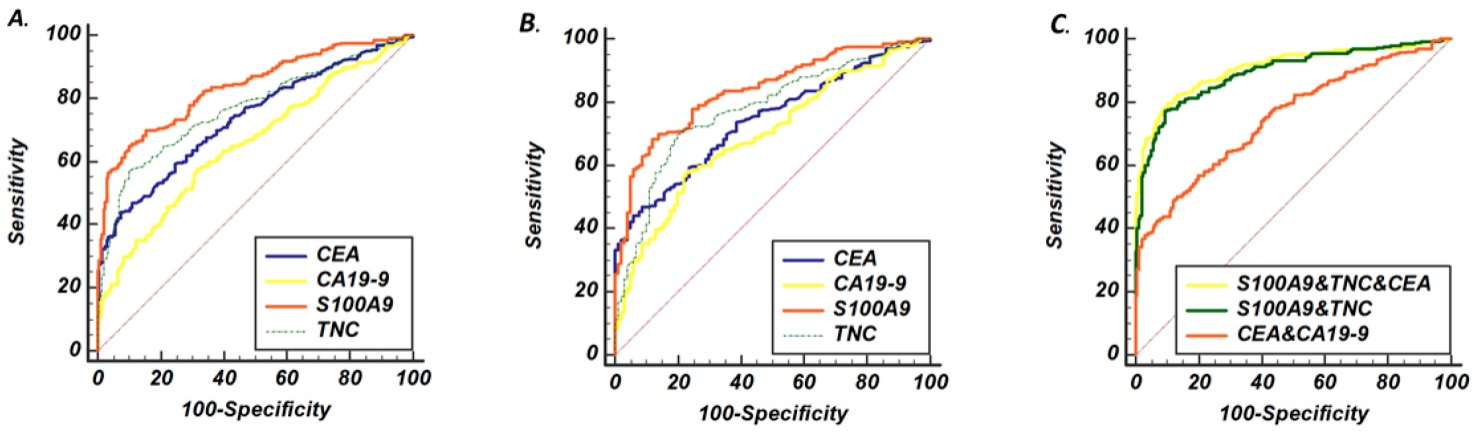
ROC curve analysis of serum concentrations from patients with CRC and controls. (A) ROC curves of S100A9, TNC, CEA and CA19-9 levels as screening biomarkers of CRC. (B) ROC curves of S100A9, TNC, CEA and CA19-9 levels as biomarkers differentiating between CRC and BCD. (C) The AUC performance of the following combinations of serum concentrations: S100A9 and TNC; CEA and CA19-9; and S100A9, TNC and CEA.

Then, to further assess the screening performance of the potential biomarkers, ROC curves were developed as shown in Fig. 2a. CEA and CA19-9 levels were used to evaluate the value of S100A9 and TNC levels as serum biomarkers in CRC screening. The S100A9 level predicted the diagnosis of CRC patients with an AUC of 0.837 (95% CI: 0.794-0.873) at a cutoff point of 18.74 ng/mL. Meanwhile, the TNC level distinguished CRC serum from healthy donor serum at a cutoff point of 3.87 ng/mL, and the AUC was 0.759 (95% CI: 0.712-0.803). The AUC value of the S100A9 level was higher than that of CEA and CA19-9 (p<0.001); the AUC value of the TNC level was also significantly different from that of CA19-9 (p<0.001).

### Performance of S100A9 and TNC levels as biomarkers differentiating between CRC and BCD

To verify the diagnostic capacity of S100A9 and TNC levels in distinguishing CRC from other benign intestinal diseases, such as colon polyps and inflammatory bowel disease, ROC curves and dot plots were constructed (Fig. 1a, b; Fig. 2b). The S100A9 and TNC concentrations in BCD patients were significantly lower than those in CRC patients (p<0.001). Similar to the results seen when comparing S100A9 and TNC levels between the CRC and HD groups, S100A9 and TNC levels showed extraordinary performance as differentiating biomarkers (AUC: 0.836 95% CI: 0.793-0.872; AUC: 0.777 95% CI: 0.730-0.819) that were better than the performance of CEA and CA19-9 levels (S100A9 vs CEA, p<0.01; S100A9 vs CA19-9, p<0.01; TNC vs CA19-9, p<0.01).

To suggest whether the S100A9 and TNC levels have the same discrimination in the diagnosis of early-stage (stages I+II) CRC, the samples from CRC patients were grouped according to the International Union against Cancer (UICC) tumor-node-metastasis (TNM) staging system, and the serum levels in patients with early-stage (stages I+II) CRC were compared to those in BCD patients.

Statistically significant higher levels of the biomarkers were found in early-stage CRC patients than in patients with BCD (p< 0.001). Interestingly, the serum S100A9 levels in CRC patients with late-stage disease were higher than those in patients with early-stage CRC (p=0.015). The TNC level also has the ability to distinguish between BCD and early-stage CRC (p<0.001). None of the data showed that the serum S100A9 and TNC concentrations of BCD patients were different from HD. As displayed in Fig. 1, all the results confirm that S100A9 and TNC levels can be used as an early diagnostic biomarker to identify CRC.

### Potential synergy between the S100A9 or TNC level and other diagnostic markers in CRC

As summarized in Table 1, we compared data at clinically recommended levels (5 or 35 ng/mL) and cutoff levels in the maximal Youden's index (Y-index) for CEA and CA19-9. We confirmed that the optimal Y-index of the S100A9 level was higher than that of the CEA or CA19-9 level in both the BCD and HD groups, and the Y-index for S100A9 and TNC levels in combination was greater than that of either one alone.

Therefore, we wanted to verify that the combination of the S100A9 or TNC level with other clinical biomarkers was able to effectively improve the ability to identify CRC from controls. A set of permutations based on these four markers and the AUC of these markers are shown in Table 2. The combination of S100A9, CEA and TNC levels demonstrated a higher AUC (0.908, 95% CI: 0.878 to 0.933) with a sensitivity of 79.8% and a specificity of 89.6%. The AUC is a much higher for the combination of S100A9 and TNC levels than the combination of CEA and CA19-9 levels (p=0.007). The combination of S100A9, TNC and CEA levels may be a better strategy for diagnosing CRC. Based on these three markers, we obtained a formula (Table 2) by using logistic regression analysis, and the cutoff point was 0.56. This formula was applicable to both the HD and BCD control groups. Using this formula, CRC may be diagnosed more accurately and easily.

### Correlationship between the S100A9/TNC serum levels and the clinical features of CRC patients

To test whether the S100A9 and TNC serum levels are influenced by other factors, we analyzed correlations between the serum biomarker concentrations and the demographic information, clinical features and tumor characteristics in CRC patients.

As shown in Table 3, gender, age and body mass index (BMI) were analyzed as demographic information, but none showed a significant correlation with serum S100A9 or TNC concentrations. In addition, we included smoking habits, drinking habits, chronic diarrhea and hemafecia as clinical features. None of the clinical features showed significant differences, but a relatively high correlation was shown between serum S100A9 levels and drinking habits (p=0.089). No correlations were found between serum S100A9 or TNC concentrations and CRC family history.

In addition, we also compared the serum S100A9 and TNC concentrations and some tumor characteristics, including the site of the lesions, tumor metastasis and tumor emboli. It is worth noting that both serum S100A9 and TNC levels exhibit a significant increase in CRC patients with tumor metastasis (p=0.025; p=0.085). The serum S100A9 concentrations also correlated with the tumor stages, as shown in Fig. 1c (p=0.015).

Additionally, diabetes was associated with S100A9 serum concentration (p=0.025). We have analyzed this result in the discussion.

### The dynamic changes in the serum S100A9/TNC levels

**Fig 3:**
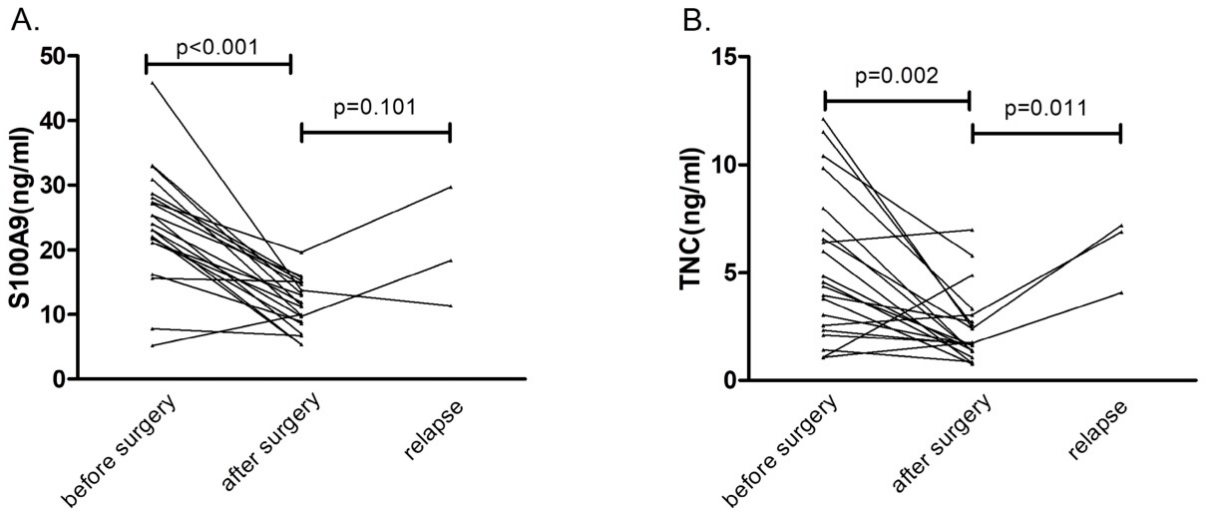
Serial serum concentrations of S100A9 and TNC. (A) Serum S100A9 levels before surgery, after surgery and during relapse in CRC patients (n=21). (B) Serum TNC levels before surgery, after surgery and during relapse in CRC patients (n=21).

In 21 patients who underwent a radical operation, the S100A9 level in serum specimens was measured before surgery (baseline) and one week after surgery by ELISA (Fig. 3). Expression levels of the serum protein biomarker were significantly lower after the operation than at baseline (p<0.001). The same result also appeared in the TNC expression pattern (p=0.002). These results suggested that serum S100A9 and TNC concentrations were derived from the tumor tissue.

Three patients with CRC relapsed during the six-month follow-up. Compared with the postoperative protein concentrations, S100A9 protein concentrations showed no obvious correlation with relapse (p=0.101), but TNC serum levels were obviously increased (p=0.011) in the three cases of patient relapse. These results may be confounded by the insufficient number of relapse cases. We will continue to follow the disease progress in the CRC patients in our study.

## Discussion

Colorectal cancer (CRC) is the third leading cause of cancer‐related deaths worldwide, and the data reveal that the overall survival rate of patients with CRC is highly dependent on the disease stage at the time of diagnosis 15. Metastasis accounts for >90% of mortality in patients with CRC 16. Unfortunately, the majority of CRC patients are diagnosed in middle- and late-stage disease. The gold standard for detecting CRC is still colonoscopy, which is expensive, especially the painless form of colonoscopy 17. Although colonoscopy increases the diagnosis rate of CRC, it is not widely available during physical examinations because it is invasive, causes discomfort, exposes patients to potential complications, and requires specific resources 18. To improve the efficiency of the early diagnosis of CRC, we hope to find one marker or a set of tumor markers that can be used in both screening and differentiating. The study of CRC diagnosis biomarkers was never stopped 19, 20. The most common biomarkers cannot meet the needs for clinical diagnosis. We selected S100A9 and TNC via proteomic analysis 9, and through our experiments, these showed better capacities to diagnose CRC than established biomarkers.

S100A9 belongs to a family of 25 homologous, low-molecular-weight, intracellular calcium-binding proteins that exhibit tissue and cell-specific expression patterns 21. S100A9 is characterized by two distinct EF-hand (helix-loop-helix) calcium-binding domains connected by a hinge region 22. Twenty-one of the human S100 genes are clustered in the chromosomal region 1q21, a region that is frequently deleted, translocated or duplicated in tumors, indicating their possible involvement in malignancy 21-23. Some studies have shown that the expression of serum S100A9 is upregulated in some cancers, such as breast cancer and pancreatic cancer 11; many other studies have found significantly increased levels of serum S100A9 in inflammatory diseases, such as rheumatoid arthritis 22. In our study, the serum S100A9 level was much higher in the CRC patient group than the control groups (BCD and HD), similar to the results of Fijneman RJ et al. 24. In concordance with Zhang X et al. 25, the Fijneman RJ et al. study revealed the mechanism by which S100A9 promoted colorectal tumorigenesis. Unlike other studies, we demonstrated that the serum S100A9 levels of BCD patients and HD were not significantly different. In many studies, the S100A9 level increases in colitis as an inflammatory protein 26, 27. Other researchers report that colonic chitinase 3-like 1 (CHI3 L1) can bind to RAGE and thus disrupt the S100A9-associated expression positive feedback loop during early immune activation, creating a S100A9 low colonic environment, especially in the later phase of colitis 28. In a future study, we will increase the sample size of inflammation patients to verify the lack of a difference in serum S100A9 concentrations between inflammation patients and healthy donors.

In our analysis, the serum levels of S100A9 were significantly correlated with diabetes, which is in agreement with Ortega FJ et al. 29. At the same time, diabetes is a risk factor for colon cancer 30. S100A9 protein functions as an inflammatory mediator that contributes to inflammation and tumor progression 25. These studies therefore provide functional evidence supporting this finding.

Some research has found that right-sided colon cancer has a worse prognosis than left-sided colon cancer 31; vascular tumor embolus also affects the prognosis of CRC patients 31. However, neither S100A9 nor TNC levels were correlated with these characteristics. In colorectal cancer patients who were diagnosed in advanced stages or with distant metastases, serum S100A9 concentrations were higher on average. In the study by Lim SY et al., S100A9 levels were also associated with tumor metastasis status 32. For CRC, 5-year relative survival can be as high as 90% or more in patients with stage I disease or as low as 10% in patients with stage IV disease 33. We suspect that CRC patients with higher S100A9 serum levels may have worse prognoses, but this needs to be validated prospectively. Paired preoperative and postoperative serum samples from 21 patients were assessed by ELISA, and the result suggested that serum S100A9 was derived from the tumor tissue. In our study, gender, age, abdominal pain and diarrhea had no effect on the concentration of S100A9. These results further illustrate that S100A9 is very stable and reliable as a diagnostic marker of colorectal cancer.

Tenascin-C (TNC) expression is involved in fetal tissue development and neoplasia in different organs, and it also facilitates the formation of cancer stroma, including desmoplasia and angiogenesis 14. In our study, TNC and CEA levels had similar efficacy as diagnostic biomarkers of CRC. It is worth noting that the TNC level showed better stability than the S100A9 level in the analysis of clinical features. The TNC level also did not correlate with tumor staging or metastasis. It is an interesting phenomenon that the serum concentration of TNC increases again in relapsing patients. However, the relationship between the prognosis of tumors and TNC expression is contradictory. In breast cancer, TNC expression in the invasive border of the tumor is regarded as a predictor of local and distant recurrence 34. It is also thought that the stromal TNC distribution has the same function in CRC 35. In contrast, it was also reported that TNC expression plays a role in preventing cancer cells from invading surrounding tissues as determined by prominent staining of TNC at cancer mesenchymal junctions and in the walls of blood vessels in the vicinity of tumors 36. In cervical and gastric cancers, the patients who have tenascin-positive tumors have a better prognosis 37. This problem is worth further exploration and validation by clinical testing.

A combination that included the levels of three serum markers, S100A9, tenascin-C and CEA, was better at detecting CRC than any one marker alone. Furthermore, considering the cost and convenience, a serum test for S100A9 and TNC levels may be a promising screening test for CRC. A concept similar to ours was applied in cholangiocarcinoma, with results showing that a combination of three serologic markers (CEA, CA125 and CA19-9) had greater sensitivity and specificity for bile duct cancer 38. The incremental benefit of the marker combination in the diagnostic prediction model can be demonstrated by the increase in the AUC. Adding CA19-9 to the model did not improve its predictive ability.

This study had some limitations. First, the number of samples in each group was not large enough, particularly in the control groups; we need to validate our findings with a larger number of patients. Second, we enrolled patients without creating a group containing patients with other colonic diseases, such as familial adenomatous polyposis and colonic adenoma. Since screening tests are usually performed in people who have already been diagnosed, our results require further validation in people with asymptomatic disease. Third, because the follow-up time was too short, our study did not include survival analysis; we will further track survival and report on it in the future. However, S100A9 serum levels had relevance to the tumor metastasis status, and TNC levels were increased in relapsed patients. Both of these results indicate that the S100A9 or TNC level may be associated with survival. Finally, although the combination of S100A9, TNC and CEA may be more sensitive and specific for CRC than the combination of CEA and CA19-9, its diagnostic efficacy may not be good enough when compared with that of colonoscopy. Therefore, additional serum tumor markers may be needed to increase the specificity and sensitivity for early diagnosis of CRC.

In general, as early diagnosis biomarkers of colorectal cancer, S100A9 and TNC levels are comprehensive. Both colon polyps and inflammatory bowel disease can progress to malignancy. Additionally, patients diagnosed with polyps and inflammatory bowel disease are advised by the NCCN Guidelines to monitor disease development periodically 30. These two serum markers can not only be diagnostic factors of CRC but also be used as long-term monitoring indexes of colonic disease. This is the first time that serum S100A9 and TNC levels in CRC patients were tested simultaneously and compared with CEA and CA19-9 levels, the combination of S100A9, TNC and CEA levels also offers a new way to diagnose CRC.

## Conclusion

In conclusion, this study aimed to assess the utility of serum S100A9 and TNC levels as non-invasive biomarkers of CRC and the probability of using them as biomarkers differentiating between CRC and benign colorectal diseases. We found relatively higher serum S100A9 and TNC levels in CRC patients. The S100A9 level was superior to the CEA level as a screening biomarker of CRC. The combination of S100A9, TNC and CEA levels was more specific and sensitive than the combination of CEA and CA19-9 levels as a diagnostic biomarker of colorectal cancer, and we created a more efficient method to diagnose colorectal cancer. However, our conclusions should be verified in further multicenter studies with larger sample sizes and more diseases.

## Figures and Tables

**Table 1:** Diagnostic values of S100A9, TNC, CEA and CA19-9 according to cutoff levels in CRC versus controls.

	CEA		CA19-9		S100A9	TNC	S100A9+TNC
CRC patients VS HDs	3.23	5	18.36	35	18.74	3.87	-
Y-index	0.35	0.3	0.2	0.16	0.56	0.5	0.72
Sensitivity (%)	44.2	33.3	35.3	18.6	64.7	57	77.5
Specificity (%)	90.3	97.1	84.5	97.1	91.3	93.2	94.2
CRC patients VS BCD patients	3.36	5	10.63	35	17.06	2.43	-
Y-index	0.39	0.31	0.35	0.16	0.56	0.5	0.64
Sensitivity (%)	44.19	33.3	57.8	18.6	68.2	70.9	81.4
Specificity (%)	94.95	98	76.8	96.9	87.9	79.8	82.8

**Table 2:** Diagnostic performance of independent or combinations of biomarkers

	AUC (95% CI)	Sensitivity (%)	Specificity (%)	Cutoff value
S100A9	0.836(0.799-0.869)	65.9	89.1	18.22
TNC	0.768(0.727-0.806)	57	90.1	3.87
CEA	0.736(0.693-0.776)	44.2	92.6	3.36
CA199	0.655(0.610-0.699)	57.8	68.3	10.63
S100A9+TNC	0.893(0.861-0.920)	77.13	90.59	_
CEA+CA199	0.751(0.709-0.790)	57	80.2	_
TNC+CEA+CA199	0.841(0.804-0.873)	77.5	81.2	_
S100A9+TNC+CEA	0.908(0.878-0.933)	79.8	89.6	_
S100A9+TNC+CA19-9	0.898(0.867-0.924)	79.1	88.1	_

p=1/{1+exp[-(-4.7175+0.28*X+0.17*Y+0.50*Z)]} x: CEA(ng/ml) Y: S100A9(ng/ml) Z:TNC(ng/ml)

**Table 3:** Associations between Serum S100A9/TNC Levels and Clinical Characteristics in CRC Patients

		n	S100A9		TNC	
			median(inter-quartile range)	p value	median (inter-quartile range)	p value
Gender	male	148(57.36%)	21.60(13.05-30.75)	0.577	4.03(2.04-5.90)	0.285
	female	110(42.64%)	22.57(15.58-28.48)		4.74(2.53-6.44)	
Age	>50	186(70.54%)	22.20(14.91-30.15)	0.77	4.52(2.20-6.05)	0.222
	≤50	72(29.46%)	22.88(14.54-28.55)		3.47(2.01-6.03)	
BMI	<18.5	28(10.85%)	25.35(15.39-38.86)	0.529	4.93(3.03-9.05)	0.194
	18.5-25	185(71.71%)	22.29(14.64-29.02)		4.27(2.12-6.01)	
	>25	45(17.44%)	21.10(16.67-30.06)		3.96(1.96-5.80)	
Smoking ^a^	Yes	77(19.84%)	21.59(12.74-30.03)	0.227	4.29(2.10-5.98)	0.849
	No	180(69.77%)	22.57(15.34-29.32)		4.29(2.14-6.23)	
Alcohol	Yes	100(38.76%)	19.86(12.94-29.29)	0.089	4.24(2.07-5.95)	0.456
	No	158(61.24%)	23.48(15.46-30.99)		4.42(2.19-6.39)	
Diabetes	Yes	22(8.53%)	22.85(14.94-30.01)	0.04	4.91(2.34-5.96)	0.716
	No	236(91.47%)	17.75(12.30-23.91)		4.11(2.11-6.09)	
Chronic diarrhea	Yes	111(43.02%)	23.77(14.77-28.70)	0.854	4.27(2.11-6.10)	0.929
	No	147(56.98%)	21.90(14.88-30.99)		4.38(2.12-6.02)	
Hemafecia	Yes	61(23.64%)	24.90(14.36-33.08)	0.204	3.44(1.87-5.79)	0.131
	No	197(76.36%)	21.91(14.88-28.74)		4.56(2.35-6.29)	
CRC in family	Yes	18(6.98%)	20.04(9.08-35.48)	0.641	3.93(1.95-5.14)	0.425
	No	240(93.02%)	22.36(14.90-29.08)		4.37(2.12-6.23)	
Site of the lesion^ a^	Right colon	61(23.64%)	21.09(13.47-25.86)	0.194	3.94(2.11-5.99)	0.619
	Left colon and rectum	163(63.18%)	22.37(14.91-30.90)		4.38(2.16-6.33)	
Tumor embolus^ a^	Yes	54(20.93%)	23.77(17.97-30.48)	0.431	4.17(2.02-5.94)	0.455
	No	144(55.81%)	22.32(14.78-30.25)		4.21(2.13-6.32)	
Stage^ a^	Ⅰ-Ⅱ	125(48.45%)	21.14(14.28-27.51)	0.015	3.90(2.02-5.99)	0.32
	Ⅲ-Ⅳ	110(42.64%)	24.92(17.65-34.76)		4.68(2.44-6.35)	
Tumor Metastasis	Metastasis	24(9.30%)	29.21(16.54-42.68)	0.025	4.92(3.43-6.85)	0.085
	non- Metastasis	234(90.70%)	22.01(14.85-28.79)		4.11(2.09-5.99)	

^a^Total numbers of cases vary among the characteristics because of missing data.

## Abbreviations

BMI: Body mass index
CI: Confidence interval
ROC: Receiver operating characteristic
ELISA: enzyme linked immunosorbent assay
CRC: colorectal cancer
TNC: Tenascin-c
HD: healthy donors
BCD: benign colonic disease
CEA: carcinoembryonic antigen
